# CASPER plus (CollAborative care in Screen-Positive EldeRs with major depressive disorder): study protocol for a randomised controlled trial

**DOI:** 10.1186/1745-6215-15-451

**Published:** 2014-11-19

**Authors:** Karen Overend, Helen Lewis, Della Bailey, Kate Bosanquet, Carolyn Chew-Graham, David Ekers, Samantha Gascoyne, Deborah Hems, John Holmes, Ada Keding, Dean McMillan, Shaista Meer, Natasha Mitchell, Sarah Nutbrown, Steve Parrott, David Richards, Gemma Traviss, Dominic Trépel, Rebecca Woodhouse, Simon Gilbody

**Affiliations:** 1 Department of Health Sciences, University of York, Seebohm Rowntree, Building, Heslington, York, YO10 5DD UK; 2 Leeds Institute of Health Sciences, University of Leeds, Charles Thackrah Building, 101 Clarendon Road, Leeds, LS2 9LJ UK; 3 Centre for Mental Health Research, University of Durham, Durham, TS17 6BH UK; 4 Research Institute, Primary Care and Health Sciences, Keele University, Keele, ST5 5BG UK; 5 Washington Singer Laboratories, School of Psychology, University of Exeter, Perry Road, Exeter, EX4 4QG UK

**Keywords:** Depression, Major depressive disorder, Older people, Elderly population, Primary care, Collaborative care, Behavioural activation, Psychosocial interventions, Randomised controlled trial, Cost effectiveness analysis, Process evaluation

## Abstract

**Background:**

Depression accounts for the greatest disease burden of all mental health disorders, contributes heavily to healthcare costs, and by 2020 is set to become the second largest cause of global disability. Although 10% to 16% of people aged 65 years and over are likely to experience depressive symptoms, the condition is under-diagnosed and often inadequately treated in primary care. Later-life depression is associated with chronic illness and disability, cognitive impairment and social isolation. With a progressively ageing population it becomes increasingly important to refine strategies to identity and manage depression in older people. Currently, management may be limited to the prescription of antidepressants where there may be poor concordance; older people may lack awareness of psychosocial interventions and general practitioners may neglect to offer this treatment option.

**Methods/design:**

CASPER Plus is a multi-centre, randomised controlled trial of a collaborative care intervention for individuals aged 65 years and over experiencing moderate to severe depression. Selected practices in the North of England identify potentially eligible patients and invite them to participate in the study. A diagnostic interview is carried out and participants with major depressive disorder are randomised to either collaborative care or usual care. The recruitment target is 450 participants.

The intervention, behavioural activation and medication management in a collaborative care framework, has been adapted to meet the complex needs of older people. It is delivered over eight to 10 weekly sessions by a case manager liaising with general practitioners.

The trial aims to evaluate the clinical and cost effectiveness of collaborative care in addition to usual GP care versus usual GP care alone. The primary clinical outcome, depression severity, will be measured with the Patient Health Questionnaire-9 (PHQ-9) at baseline, 4, 12 and 18 months. Cost effectiveness analysis will assess health-related quality of life using the SF-12 and EQ-5D and will examine cost-consequences of collaborative care.

A qualitative process evaluation will be undertaken to explore acceptability, gauge the extent to which the intervention is implemented and to explore sustainability beyond the clinical trial.

**Discussion:**

Results will add to existing evidence and a positive outcome may lead to the commissioning of this model of service in primary care.

**Trial registration:**

ISRCTN45842879 (24 July 2012).

## Background

### Problem to be addressed

Depression accounts for the greatest burden of disease among all mental health problems and is expected to become the second-highest of all general health problems by 2020 [1]. Depression is common among older people and people with co-morbid chronic medical conditions and cognitive impairment, and can result in poor quality of life [2]. Although 10% to 16% of people aged over 65 years may experience depressive symptoms, with 2% to 4% meeting formal diagnostic criteria for moderate to severe depression, [3] the condition is under-diagnosed and often inadequately treated in primary care [4].

Older people with a long-term condition are five times more likely to suffer depression. For example, 50% of people with Parkinson’s disease will suffer depression, 25% following stroke and 20% with coronary heart disease, 24% with neurological disease and 42% with chronic lung disease [2]. Beyond personal suffering and family disruption, depression worsens the outcomes of many medical disorders and promotes disability [2].

The impairments in quality of life associated with depression are comparable to those of major physical illness and among older people a clinical diagnosis of major depression is the strongest predictor for impaired quality of life (QoL) [5]. Demographic projections indicate a growing ageing population which calls for effective strategies to specifically tackle depression in older people [6]. However, an important factor noted by current and previous studies [7–9] is the complex nature of identifying and treating depression in an ageing population.

### The need for a trial

#### Management of depression in older people

The vast majority of depression in older people can be managed entirely in primary care, without recourse to specialist mental health services [10–12]. A range of individual treatments has been shown to be effective in older people, including anti-depressants and psychosocial interventions [10], but a repeated observation among all trials of people with depression has been the failure to integrate these effective elements of care into routine primary care services [13].

Current NICE guidelines recommend a stepped care framework for the management of depression. Essentially, in stepped care, the ‘least intrusive, most effective intervention’ is provided first; if this is not beneficial, or if it is declined, patients should be offered an intervention from the next step [14].

However, in the UK, depression in older people is under-diagnosed and under-treated [15]. Even when recognised, the provision of psychosocial interventions for this age group is inadequate. For example, there has been minimal provision of psychological treatment for older people under the Improving Access to Psychological Therapies (IAPT) programme [16].

Despite GPs being encouraged through the Quality and Outcomes Framework (QOF) to case-find for depression in older people, there is little evidence that this has translated into better management or identification for this disorder [11]. This indicator in the QOF framework has now been removed.

### Collaborative care for older adults

Collaborative care is a complex intervention delivered in primary care, based on chronic disease management models used for conditions including diabetes. This model of care, also referred to as case management, facilitates the delivery of effective forms of treatment (such as pharmacotherapy and/or brief psychological therapy) [17]. A widely-accepted definition established by Gunn [18] states that collaborative care incorporates four related criteria: a multi-professional approach to patient care; a structured management plan; scheduled patient follow-ups; and enhanced communication between professionals.

There is evidence demonstrating the effectiveness of collaborative care with behavioural activation in improving treatment of depression for adults over 60 in the United States [7, 19] and a US systematic review of 37 randomised trials comparing collaborative care interventions with usual care reported significant improvements of the quality of depression care and patient outcomes, lasting up to 2 to 5 years, along with increased medication adherence and improved satisfaction among patients and primary care physicians [20]. However, the transferability of this model of care to the UK NHS cannot be assumed and requires independent evaluation.

Until recently, there has been limited evidence of the effectiveness of collaborative care for depression in the UK. In 2013 a UK trial of collaborative care for a general adult population (CADET) reported evidence of persistent positive effects on symptoms of moderate to severe depression among adults aged 18 years and over, up to 12 months after the start of the intervention. However, this trial did not collect specific data on older people [8]. The CASPER Plus trial intervention has been developed specifically for older people and, by increasing follow up to 18 months, aims to extend the evidence base for this population.

Despite recent investment under the Improving Access to Psychological Therapies (IAPT) initiative, the capacity for specialist mental health services to provide collaborative care is constrained and demand would quite quickly outstrip supply. Hence any feasible strategy will be both low intensity and offered within primary care [21, 22]. The ubiquity of depression in primary care settings and the poor integration and/or co-ordination of care have led to strategies to re-engineer the delivery of care.

In a *BMJ* editorial (2004) on the management of depression in older people Chew-Graham notes ‘Innovations in the management of depression have been evaluated. The best results come from models that use multifaceted interventions and principles of collaborative care’ [6].

In our own trials of collaborative care in working age adults, we have shown positive outcomes through the combination of medication management and a low intensity psychological intervention, ‘behavioural activation’ (BA), which can be readily delivered in eight to 10 sessions by a trained case manager either over the phone or face to face for those who have difficulty using or accessing phone-based therapy [23]. Briefly, behavioural activation focuses on the behavioural deficits common among those with depression, it reintroduces positive reinforcement and reduces avoidance. The effectiveness of BA is now well demonstrated in trials and has been shown to be at least as effective as more complex forms of psychological intervention such as cognitive behavioural therapy [24]. Several trials have shown positive results in older adult populations and the focus on self-enhancement and physical activity is especially relevant [25].

### The CASPER plus trial

CASPER Plus is a multi-centre, randomised controlled trial of a collaborative care intervention for individuals aged 65 years and over experiencing moderate to severe depression. For a detailed description see Table 1 ‘The SPIRIT checklist’.

**Table 1:** **The SPIRIT checklist**

Data category	Information
Primary registry and trial identifying number	ISRCTN 45842879
Date of registration in primary registry	24 July 2012
Secondary identifying numbers	HTA - Project: 10/57/43 CASPER Plus
Source of monetary of material support	National Institute of Health Research Health Technology Assessment (NIHR HTA)
Primary sponsor	University of York
Contact for scientific and/or public queries	Professor Simon Gilbody 01904 430000 (simon.gilbody@york.ac.uk)
Public title	The CASPER Plus study
Scientific title	Collaborative Care for Screen-Positive Elders with major depressive disorder
Countries of recruitment	UK
Health condition(s) or problem(s) studied	Depression in older people
Intervention(s)	Behavioural activation (BA) and medication management delivered in a collaborative care framework by a case manager liaising with general practitioners/health professionals/third sector, with supervision from a mental health specialist
Key inclusion and exclusion criteria	**Inclusion criteria:** Aged 65 years and over; screen positive to at least one of the Whooley questions and who, on further assessment with the MINI diagnostic tool and PHQ-9 questionnaire, have DSM-IV Major Depressive Disorder (MDD). See protocol paper
	**Exclusion criteria:** Known alcohol dependency (as recorded on GP records); any known co-morbidity that would in the GP’s opinion make entry to the trial inadvisable (for example, recent evidence of self-harm, known current thoughts of self-harm, significant cognitive impairment); other factors that would make an invitation to participate in the trial inappropriate (for example, recent bereavement, terminal illness); known to be experiencing psychotic symptoms (as recorded on GP records)
Study type	Randomised controlled trial
	Interventional
	Allocation: randomised
	Masking: none
	Primary purpose: prevention and/or improvement of symptoms
Date of first recruitment	15 September 2012
Target sample size	450
Recruitment status	Recruiting
Primary outcome(s)	Depression severity at 4 months (following intervention) by self-report using the Patient Health Questionnaire 9 (PHQ-9) on a continuous scale. We will also measure outcome at 12 and 18 months using the PHQ-9 to examine any sustained impact of the intervention
Secondary outcome(s)	Secondary outcomes include the SF-12 and GAD-7 at 4, 12 and 18 months. We will also collect data on somatic symptom severity using the PHQ-15, participant resilience using the CD-RISC2 and cost effectiveness including the EQ-5D, prescribed medication and health and social care use. See protocol paper for references

### Research objectives

1. To establish the clinical effectiveness of a collaborative care intervention for older people with screen-positive above-threshold (‘major depressive disorder’) depression within a definitive RCT.
2. To examine the cost effectiveness of a collaborative care intervention for older people with screen-positive above-threshold (‘major depressive disorder’) depression within a definitive RCT.

## Methods/design

The CASPER Plus trial aims to evaluate the clinical and cost effectiveness of collaborative care in addition to usual GP care versus usual GP care alone. The trial is designed as a multi-centre, un-blinded, pragmatic [26], randomised controlled trial lasting 42 months, comprising an 18-month definitive trial, an 18-month follow-up phase, followed by 6 months for analysis and final report. Ethical approval from the National Health Service Research Ethics Committee (NHS REC) has been obtained from Leeds East REC (reference 10/H1306/61) and local approvals have been gained through local NHS R & D offices: Leeds PCT, North Yorkshire & York PCT; East Riding of Yorkshire PCT; Newcastle PCT and Hull Teaching PCT; County Durham and Tees Valley PCT; Northumberland Care Trust; Northumberland Tyne and Wear NHS Trust.

### Study design

**Figure 1:**
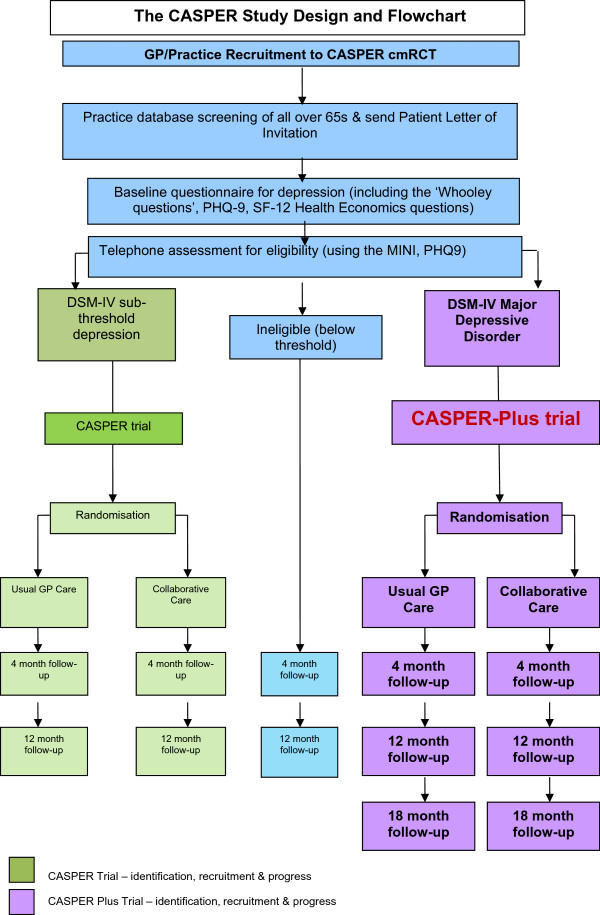
**Design and flowchart of the CASPER study.**

The trial is a sub study to the wider CASPER study [27] which commenced in 2011 and has successfully recruited an epidemiological cohort of people aged 65 years and over (the CASPER cohort) identifying those eligible to participate in a trial of collaborative care for sub-threshold depression (the CASPER trial). As part of a simultaneous recruitment process, the CASPER Plus trial will identify participants with major depressive disorder. A flowchart of the CASPER study, outlining this process, is detailed in Figure 1.

### CASPER plus trial recruitment

#### Recruitment of practices

GP practices will be recruited following introductions by the local PCRN. Practices will be paid a set fee by the CLRN for conducting a search of patients who meet the inclusion and exclusion criteria, plus a further reimbursement for each person invited and for the collection of objective data at the end of the trial.

### Participant recruitment

Participants will be recruited from four main recruitment centres in the North of England: York (the trial centre), Leeds, Durham, Newcastle and their surrounding areas. Participants to the CASPER Plus trial are identified via GP practices. Informed consent will be gained from each participant.

Recruitment methods include direct referral by GPs of patients who consult for depression and targeted searches in order to identify those aged 65 and over most at risk of depression, for example, those with chronic conditions such as diabetes, CHD and COPD. All patients who have been identified by their GP practice as eligible to join the study are then screened by individual GPs before being sent an invitation pack; if referred by their GP, patients are given the pack at consultation. Patients who wish to take part in the CASPER study are asked to return their completed consent and background information forms by post to the study centre. All consenting participants are then sent a baseline questionnaire by the research team and asked to complete and return this. Inclusion in the CASPER Plus trial is dependent on participants meeting the inclusion criteria and currently experiencing major depressive disorder.

### Identifying participants with major depressive disorder

On receipt of a valid baseline questionnaire, all participants are contacted over the telephone by a trained researcher and a diagnostic interview is carried out. The major depressive episode module of the Mini International Neuropsychiatric Interview (M.I.N.I) is used to ascertain the presence or absence of depressive symptoms and depressive disorders. All eligible participants diagnosed with major depressive disorder are randomised to either the intervention group: collaborative care with behavioural activation, medication management and active surveillance; or the control group: usual primary care management of above-threshold depression (major depressive episode) offered by the patient’s GP, in line with NICE depression guidance and local service provision [10, 28] Table 2 shows the criteria for identifying a major depressive episode using the DSM-IV. Patients are randomly allocated by the York Trials Unit automated system through the trial database. Participants are sent a letter indicating the group to which they have been randomly allocated; GPs are sent a similar letter notifying them of their patients’ allocation.

**Table 2:** **Diagnostic criteria for depression based on DSM-IV**

**Based on the nine-item depression module from the MINI participants are classified in the following way:**	
• ***Major depressive episode:*** Five or more symptoms, including one of the key symptoms	
• ***Sub-threshold depressive symptoms:*** Two to four symptoms, may or may not include a key symptom	
• ***Non-depressed:*** None or one symptom	
**Symptoms:**	
1.	Depressed mood^a^
2.	Loss of interest^a^
3.	Significant weight loss or gain or decrease or increase in appetite
4.	Insomnia or hypersomnia
5.	Psychomotor agitation or retardation
6.	Fatigue or loss of energy
7.	Feelings of worthlessness or excessive or inappropriate guilt
8.	Diminished ability to think or concentrate, or indecisiveness
9.	Recurrent thoughts of death, recurrent suicidal ideation without a specific plan, or suicide attempt or a specific plan

^a^Key symptom.

### Inclusion and exclusion criteria

Eligible participants will be identified from practice lists at participating GP practices.

The following eligibility criteria will be used:

### Inclusion criteria

-  Aged 65 years and over
-  Screen-positive to at least one of the Whooley questions [29, 30] listed below and who, on further assessment with the MINI diagnostic tool and PHQ-9 questionnaire have DSM-IV Major Depressive Disorder (MDD) [31, 32].

### The Whooley questions

1. ‘Over the past month, have you been bothered by feeling down, depressed or hopeless?’
2. ‘Over the past month, have you been bothered by having little interest or pleasure in doing things?

### Exclusion criteria

Participants are excluded according to the GP’s discretion, during screening of the patient list, using the following guidelines:

-  Known alcohol dependency (as recorded on GP records).
-  Any known co-morbidity that would in the GP’s opinion make entry to the trial inadvisable (for example, recent evidence of self-harm, known current thoughts of self-harm, significant cognitive impairment).
-  Other factors that would make an invitation to participate in the trial inappropriate (for example, recent bereavement, terminal illness).
-  Known to be experiencing psychotic symptoms (as recorded on GP records).

### Trial intervention

#### Intervention group: collaborative care with behavioural activation

Participants randomised into the intervention group will receive collaborative care (including BA) with medication monitoring and management. This experimental intervention is a bespoke collaborative care designed and delivered specifically for those aged 65 years or over with above threshold, case-level depression over eight to 10 weekly sessions. Participants randomised to the intervention group will be contacted by the allocated case manager to arrange their first session face to face.

Collaborative care will be delivered by a case manager (CM: a primary care mental health worker) within a ‘stepped care framework’ , such that those whose depression deteriorates are ‘stepped up’ from low intensity care to a more intensive form of management including medication monitoring. This will be delivered according to an established protocol [33].

The five core components of the intervention are described below:

1. Patient-centred assessment and engagement: patients are usually first assessed in their own residential setting. The severity of depression and associated behavioural and social deficits are assessed. The presence of depressive symptoms and behavioural deficits are described and patient information materials are given.
2. Symptom measurement and monitoring: a standardised assessment of symptom severity is made by symptom tracking (to judge response, failure to respond or deterioration) using the GDS-15, a reliable and valid measure for depressive symptom severity in older adults, which is then carried out at all subsequent patient contacts.
3. Medication management: the prescription of anti-depressant medication is entirely at the discretion of the general practitioner. We will encourage GPs to consider NICE guidance in their prescribing decisions. The concordant use of medication by patients will be encouraged by the case manager if a prescription has been initiated by the GP. Patient concerns (such as ‘addiction’) and non-compliance will be explored and addressed during sessions. There will be active liaison with GPs to encourage follow-up patient appointments with the GP if poor adherence is noted by the CM.
4. Active follow-up: all patients are proactively followed up by the CM for 8 weeks using face-to-face meetings or telephone contacts. Our own experience is that telephone contacts are acceptable and that patients can be engaged using this means of communication [33]. We have adapted this means of delivery to the specific needs of people aged 65 years and over.
5. Delivery of Behavioural Activation (BA): patients are offered the option of BA delivered over eight sessions by their case manager. BA consists of a structured programme of reducing the frequency of negatively reinforced avoidant behaviours in parallel with increasing the frequency of positively reinforcing behaviours to improve functioning and raise mood. During this time patients will remain under the medical care of their general practitioner. We have demonstrated that BA is potentially effective in older adults [25] and have recently demonstrated the effectiveness of this approach in working age adults [34]. Following completion of the intervention, the participant is offered a final contact 6 weeks later. This enables the participant to consolidate new information learned and reflect on any behavioural changes made. If during treatment it becomes apparent that symptoms are not improving the case manager and participant will collaboratively discuss options for further treatment. This may require referral back to the GP for possible medication review or prescription or stepping up into the local Improving Access to Psychological Therapies (IAPT) service for interventions such as Cognitive Behavioural Therapy or Counselling.

As part of collaborative care the GP is informed routinely by letter at the beginning, mid-point and end of the participant’s progress. If the CM is concerned about the participant’s mental wellbeing it may be necessary, with the participant’s consent, to contact their GP to discuss these concerns. The only time consent may not be required is if the participant’s life is at risk, from themselves or others.

The additional elements of collaborative care include: telephone support; symptom monitoring and active surveillance (facilitated by a computerised case management system PC-MIS); medication monitoring and a low intensity psychosocial intervention (behavioural activation). PC-MIS is a web-based management information system developed collaboratively by the Department of Health Sciences Mental Health Research group and Health Sciences IT Services, with the specific purpose of managing high-volume mental health patients, through stepped care. It allows assigned clinicians and supervisors to access participant details and their current treatments, thereby improving clinical and information governance.

### Intervention manual

All CMs follow the guidance of a detailed intervention manual, thereby optimising consistency across all the sites. The work of CMs is overseen by an older persons’ mental health specialist (old age psychiatrist or psychologist). This is made possible by each CM being assigned a supervisor from the principle site (University of York) who has completed the IAPT Psychological Wellbeing Practitioner supervisor course.

### Usual care group

Participants allocated to the usual care group will receive usual primary care management of case level depression offered by their GP, including the prescription of any necessary medication. Participation in the trial will therefore not affect medication prescribing, which will remain entirely in the control of GPs.

### Case manager training

All CMs will participate in a comprehensive 3-day training programme delivered by supervisors at the University of York. This includes training on the use of the study specific manual, procedures, behavioural theories underpinning BA, medication management and specific training on working with older adults. This in-depth training aims to ensure adherence and fidelity to the model. We will not formally assess adherence and competence.

### Sample size calculation

In line with the IMPACT US trial [7] and the point estimate from our UK pilot trial [35], a standard effect size of 0.35 is sought as a conservative estimate of the detectable difference in mean PHQ-9 [36] depression severity scores between collaborative care and usual care. This difference equates to approximately 1.4 PHQ-9 score points (assumed SD = 4.1). In order to detect such a difference with 80% power at the 5% two-sided significance level, assuming an ICC of 0.02 (case load size of 20) and 20% loss to follow-up, the sample size needed will be 450 (225 in each group).

### Follow-up

Data collection will occur at six time points: at invitation, baseline (pre-randomisation/pre-assessment), diagnostic interview for participants entering the trial, at 4 months, 12 months and 18 months post randomisation/assessment. Additionally, primary care sources will be checked for medication prescribing during the participant’s time in the trial. Postal questionnaires will be used for data collection at baseline, 4-, 12- and 18-month follow-ups.

### Outcome measures

The primary outcome measure is mean depression severity at 4 months by self-report using the Patient Health Questionnaire 9 (PHQ-9) on a continuous scale. We will also measure outcome at 12 and 18 months using the PHQ-9 to examine any sustained impact of the intervention.

Secondary outcomes include the SF-12 [37] and GAD-7 [38] at 4, 12 and 18 months. We will also collect data on somatic symptom severity using the PHQ-15 [39], participant resilience using the CD-RISC2 and cost effectiveness including the EQ-5D [40], prescribed medication and health and social care use. Resource utilisation and cost data will be collected to fully reflect the management of depression and the consequential ‘total healthcare costs’ for individuals in both collaborative care and usual care groups, and these will be analysed within a societal perspective.

### Data analysis

#### Statistical analysis of clinical data

We will analyse the data on an ‘intention-to-treat’ basis. The primary outcome of depression severity is the continuous PHQ-9 depression score. Data will be analysed by a mixed effects model to compare collaborative care with usual care over all follow-up time points. The analysis will be adjusted for baseline depression severity (PHQ-9 score) and physical functional limitations (SF-12 physical component score). Estimates of the mean difference between randomised groups at 4 months follow-up will constitute the primary end point and will be presented with 95% confidence intervals. The analysis will include all patients with valid PHQ-9 scores at any follow-up time point and complete covariate data [30].

The number of non-responders will be calculated for each treatment group and response rates compared. We will undertake a secondary analysis to explore the impact of missing data by including predictors of non-response as covariates in the primary model. Analyses of secondary outcomes will be conducted using linear or logistic mixed models, depending on the outcome measure, adjusting for the same covariates as the primary analysis. Variability of within-therapist clustering will be explored by including CMs as random effects in the primary analysis. Full details will be provided in the statistical analysis plan.

#### Analysis of economic data

Economic evaluation will take the form of within-trial cost-utility analysis to determine the incremental cost per quality adjusted life year (QALY) for treatment with collaborative care against usual care in individuals with depression. Following NICE evaluation guidelines, the primary analyses will be conducted from the perspective of the UK NHS and personal and social services (PPS). The cost-effectiveness acceptability curve (CEAC) will represent the probability that collaborative care is cost-effective compared to usual care for a range of maximum monetary values (ceiling ratios) that a UK decision-maker may be willing to pay for an increase in one unit of QALY. Furthermore, a net benefit analysis will be undertaken to evaluate the net monetary gain that can be achieved with implementation of collaborative care thus indicating to decision makers the value of the intervention in terms of monetary gain.

### Process evaluation

While a considerable evidence base exists for the role of collaborative care in improving treatment of depression in the USA, there is a recognised gap between demonstrated efficacy in trials and implementation in everyday practice, with particular uncertainty around whether the model will effectively translate to UK healthcare systems. The UK Medical Research Council has highlighted the need for process evaluations to understand the problems of integrating interventions into healthcare settings. A recent trial of collaborative care in the adult population [41] highlighted organisational barriers to embedding the intervention into routine primary care. The process evaluation for CASPER Plus will add to the existing literature by including data collection from patients, as well as identifying barriers and facilitators to implementation of this model of care in the older population, who view ‘depression’ very differently from the younger adult population [4].

We will therefore undertake a qualitative process evaluation to evaluate the extent to which the collaborative care model was delivered and received, and how it impacted on practices. The aims of the process evaluation are to explore:

1. Feasibility and acceptability of the collaborative care intervention as experienced by both patients and professionals (CMs and GPs).
2. Likely sustainability of collaborative care models of care beyond the trial.

The COREQ checklist, consolidated criteria for reporting qualitative research, is given in Table 3.

**Table 3:** **The COREQ checklist**

Item	Description
Domain 1: Research team and reflexivity	
Interviewer	Researchers KB and KO will conduct the interviews
Credentials of interviewers	KB MSc Environmental Epidemiology and Policy
	KO MA English Literature
Occupation of interviewers	Researchers based in Department of Health Sciences, Seebohm Rowntree Building, University of York, Heslington, York YO10 5DD, UK
Gender of interviewers	Female
Training and experience	Researchers conducting interviews have undergone basic training in qualitative methods, and will be closely supervised and mentored by CCG/SG
Relationship with patients	Researchers may have conducted a baseline interview with patients prior to qualitative interview, or may not have met or spoken to the interview participant before
Participant knowledge of the interviewer	The research and purpose of the interview will be explained as part of the consent process with the participant
Interviewer characteristics	Interviewers conducting the qualitative study are members of the research team for the CASPER Plus study, thus potentially biased in their view of the intervention and trial
Domain 2: Study design	
Theoretical framework	The qualitative exploration has some *a priori* assumptions; an initial thematic analysis using principles of constant comparison will be followed by analysis using the Normalisation Process Theory [42] in order to explore how the intervention might be incorporated into routine practice
Sampling of participants	A purposive sample of patient participants will be invited to be interviewed, ensuring variation in age (65 to 79 years and 80+ years), gender, research site. We invite patients who have completed the intervention and those who decline to participate in the trial and those who have ‘dropped out’
	All CMs will be invited to be interviewed
	All GPs in participating practices will be invited to be interviewed, and sampling will ensure a mix of age, years in practice, gender, demography and size of practice
Method of approach	Potential patient participants were invited by mail which included a letter, information leaflet and consent form
	CMs were approached either directly or by email with information leaflet and consent form
	GPs were contacted by email or letter, with attached information leaflet and consent form
Sample size	Sample size of CM data set will be limited by number of CMs in the trial
	Interviews with patient participants and GPs will continue until category saturation is achieved in each data set
Non-participation	We will record how many potential participants who were invited declined to participate
Setting	CMs and GPs will be interviewed in their place of work
	Patient participants will be offered a choice of venue for the interview: home visit, GP practice, university office, other venue of their choice
Presence of non-participants	For patient participants, it is possible that spouses or carers may be present during the interviews
Domain 3: Data collection	
Interview guide	The interview guide will be developed by the research team with reference to their previous work, the wider literature and through discussion of the study’s aims and objectives
Repeat interviews	No repeat interviews are planned
Recording	Interviews will be audio-recorded, downloaded and transcribed (anonymising the data at this point). The digital recording will be deleted
Field notes	Field notes will be kept by the interviewers, and discussed in research meetings. These notes will contribute to modification of the interview guide and to data analysis
Duration	A record of the duration of each interview will be kept
Data saturation	Interviews will be conducted until data saturation is achieved in each data set
Transcripts returned	We do not plan to send the transcripts to participants for comment
Domain 4: Analysis and findings	
Number of data coders	Three researchers (KO, KB, CCG) will conduct data coding, with discussion of the coding at regular research meetings
Description of the coding tree	We do not plan to publish a description of the coding tree
Derivation of themes	Some of the themes will be *a priori* themes, anticipated from the wider literature; we anticipate that new themes will emerge from analysis of the data, specific to this age group of patients
Software	Software will not be used to organise the data
Participant checking	We anticipate that there may be occasional instances when we need to re-contact the participant to clarify some point in the transcript
Reporting - Quotations	We will use illustrative data extracts to support our findings
Data consistency	We will look for dis-confirmatory evidence as we conduct interviews and analysis. We will result such evidence
Clarity of major themes	We will present the major themes in any publications
Clarity of minor themes	We will present minor themes and dis-confirmatory evidence in our outputs, particularly in report to HTA

### Data collection

Interviews will be conducted by a qualitative researcher with all CMs, a sample of trial participants including those who declined the intervention or dropped out, and a sample of GPs. The interviews will be digitally-recorded (with participant’s consent) and transcribed, and transcripts will form the data to be analysed initially thematically, using a constant comparison approach. The topic guide will be modified as data collection and analysis progresses. Data analysis will involve a process of organising the data, descriptive coding, interpretive coding, writing and theorising.

Comparative analysis both within and across the data sets will be carried out to allow data from different participants to be compared and contrasted. Deviant cases will be actively sought throughout the analysis and emerging ideas and themes modified in response. Data analysis will involve at least two members of the research team to independently read transcripts and discuss coding and emerging themes, supervised by CCG. We will then reanalyse the data using the framework of the Normalisation Process Theory [42] in order to explore the barriers and facilitators of incorporating this intervention into routine care.

The process evaluation is an integral component of CASPER Plus [43] and will add value to the trial in explaining the findings and increasing the utility of the trial results and implications for clinical practice.

## Discussion

Recruiting participants from this population may prove to be challenging and we are mindful of the need to adapt our recruitment methods. Adjustments have been made in our search criteria to account for the difference in population groups, that is, older adults with major depressive disorder compared to those with sub threshold depression, and a larger invitation base will be necessary in order to meet our recruitment target. We anticipate that this study will add to the existing international knowledge base of collaborative care for depression which has not yet been explored for an older population in a UK setting. In our economic analysis we aim to provide evidence of whether this intervention for older people will be cost effective when delivered within primary care. [44] We hope results will translate successfully into both health policy and practice.

### Patient and public involvement

In advance of recruitment to the CASPER Plus trial, a patient and public involvement group was established. In an effort to meet NICE guidelines for patient and public involvement, to enable transparency and improve relevance of the study for the public, members of this group were asked to review and comment on the invitation materials.

### Trial management

The Chief Investigator, Simon Gilbody, is responsible for the overall management of the CASPER Plus study. The York based Trial Manager, Helen Lewis, will be responsible for the co-ordination of the study between the sites. Recruitment has been extended from two sites - originally York (PI Simon Gilbody) and Leeds (PI John Holmes) - to four, with the addition of Durham (PI David Ekers) and Newcastle (PI Esther Cohen-Tovee) with the aim of meeting the recruitment target for this difficult to reach group. A trial research team in each site will carry out the day to day activities involved in recruiting and running the trial. Delivery of collaborative care is carried out by dedicated, skilled and trained CMs at each site, who are specifically trained for the trial and supervised by trial supervisors to ensure continuity of delivery. The process evaluation will be managed by Carolyn Chew-Graham.

A Trial Management Group oversees the operational management of the CASPER Plus trial. Additionally, an independent Trial Steering Committee and Data Monitoring & Ethics Committee have been established; both committees meet at regular intervals to oversee the overall management of this trial. Further information on membership can be obtained from the corresponding author, Professor Simon Gilbody.

For further information on the HTA’s projects, see: http://www.nets.nihr.ac.uk/programmes/hta.

## Trial status

The CASPER Plus trial is still in its recruitment phase. The process evaluation has begun.

## Abbreviations

BA: Behavioural activation
CASPER: Collaborative care in screen positive elders
CBT: Cognitive behavioural therapy
CC: Collaborative care
CHD: Coronary heart disease
CLRN: Clinical research network
CM: Case manager: a primary care mental health worker
COPD: Chronic obstructive pulmonary disease
EQ-5D: Measure of health outcome from the EuroQol group
GAD-7: Generalised anxiety disorder assesment-7
GDS-15: Geriatric depression scale-15
IAPT: Improving access to psychological therapies
MDD: Major depressive disorder
MINI: Mini International neuropsychiatric interview
PC-MIS: Patient case management information system
PCRN: Primary care research network
PHQ9: Patient health questionnaire-9
QOF: Quality and outcomes framework
QoL: Quality of life
SF-12: Short form health survey-12.
