# Erratum to: CASPER plus (CollAborative care in screen-positive EldeRs with major depressive disorder): study protocol for a randomised controlled trial

**DOI:** 10.1186/s13063-016-1361-x

**Published:** 2016-04-27

**Authors:** Karen Overend, Helen Lewis, Della Bailey, Kate Bosanquet, Carolyn Chew-Graham, David Ekers, Samantha Gascoyne, Deborah Hems, John Holmes, Ada Keding, Dean McMillan, Shaista Meer, Jodi Meredith, Natasha Mitchell, Sarah Nutbrown, Steve Parrott, David Richards, Gemma Traviss, Dominic Trépel, Rebecca Woodhouse, Simon Gilbody

**Affiliations:** Department of Health Sciences, University of York, Seebohm Rowntree, Building Heslington, York, YO10 5DD UK; Leeds Institute of Health Sciences, University of Leeds, Charles Thackrah, Building, 101 Clarendon Road, Leeds, LS2 9LJ UK; Centre for Mental Health Research, University of Durham, Durham, TS17 6BH UK; Research Institute, Primary Care and Health Sciences, Keele University, Keele, ST5 5BG UK; Washington Singer Laboratories, School of Psychology, University of Exeter, Perry Road, Exeter, EX4 4QG UK

## Erratum

After publication of this work [1], we noted that we inadvertently failed to include the complete list of all coauthors. The full list of authors has now been added, and the Authors' contributions and Competing interests section modified accordingly. We are publishing this erratum to update the author list, which is as follows: Karen Overend, Helen Lewis, Della Bailey, Kate Bosanquet, Carolyn Chew-Graham, David Ekers, Samantha Gascoyne, Deborah Hems, John Holmes, Ada Keding, Dean McMillan, Shaista Meer, Jodi Meredith, Natasha Mitchell, Sarah Nutbrown, Steve Parrott, David Richards, Gemma Traviss, Dominic Trépel, Rebecca Woodhouse and Simon Gilbody.
